# Associations between red blood cell count and metabolic dysfunction-associated fatty liver disease(MAFLD)

**DOI:** 10.1371/journal.pone.0279274

**Published:** 2022-12-27

**Authors:** Xinyi Dai, Guowei Zhou, Luzhou Xu

**Affiliations:** Affiliated Hospital of Nanjing University of Chinese Medicine, Jiangsu Province Hospital of Chinese Medicine, Nanjing, China; Bolu Abant İzzet Baysal University: Bolu Abant Izzet Baysal Universitesi, TURKEY

## Abstract

**Background:**

Some studies found that red blood cell count (RBC) was an unrecognized risk factor for non-alcoholic fatty liver disease (NAFLD). While the epidemiological data underpinning the evidence is very limited. As there are some differences between the latest criteria of metabolic dysfunction-associated fatty liver disease (MAFLD) and NAFLD, itis necessary to evaluate the relationship between RBC and MAFLD.

**Methods:**

We performed a cross-sectional analysis of the National Health and Nutritional Examination Survey (NHANES)2017-2018 cohort, including 4477 participants. Hepatic steatosis was determined when the value of controlled attenuation parameter (CAP) obtained by Fibroscan was ≥274 dB/m. Multivariate logistic regression analysis was used to estimate the association between RBC and MAFLD. We estimated the adjusted odds ratio (OR) of RBC for MAFLD, and the nonlinear relationship between RBC and MAFLD was further described using smooth curve fittings and threshold-effect analysis.

**Results:**

We found that MAFLD risk was significantly higher according to RBC quartiles. The adjusted odds ratio (OR) and 95% confidence intervals (CIs)for the highest RBC quartile were 1.5(1.0, 2.3) for male and 1.1 (0.8, 1.6) for female, respectively. As for male, a non-linear relationship was discovered between RBCs and MAFLD, with a RBC threshold of 4.2. The effect sizes and confidence intervals on the right side of the inflection point were 1.5 (1.0, 2.0) (P for nonlinearity = 0.027). The sensitivity analysis showed a similar result.

**Conclusion:**

We demonstrated that that elevated RBC level is associated with the higher risk of MAFLD in male. The positive relationship was not significant in females after full adjustment. Our finding provided novel evidence indicating that RBCs might be a potential biomarker for MAFLD.

## Introduction

About a quarter of the global population suffered from fatty liver, which was frequently accompanied by metabolic dysfunction and there was no approved medication therapy for this disease. Experts agreed that metabolic dysfunction-associated fatty liver disease (MAFLD) can be a a better general term as patients with nonalcoholic fatty liver disease (NAFLD) are usually heterogeneous [1]. The MAFLD population shows higher liver enzyme levels as well as more lipid and glucose metabolism related diseases. As an revised definition of MAFLD can particularly identify more at risk populations in clinical practice as the metabolic syndrome dramatically raised the risk of fibrosis, cirrhosis, and hepatocellular carcinoma [2]. Metabolic disorder is key to the diagnosis of MAFLD, however, patients who consumed alcohol or had other chronic liver diseases were not excluded from the diagnosis [3]. MAFLD and NAFLD differ significantly in prevalence, risk factors, and all-cause mortality [4]. As MAFLD will take the role of NAFLD in the nomenclature transition, further research into the disease’s intricate pathophysiology is required.

Erythrocytes not only reversibly transport oxygen and carbon dioxide mainly by hemoglobin(Hb), but also contain a variety of additional proteins, with many functions in modulating oxygen transport, vascular cell communication, and inflammation being discovered [5]. Previous studies showed that RBC is associated with insulin resistance and metabolic syndromes(Mets),suggesting it might be served as a potential predictor of Mets risk [6–8]. Researchers found that individuals with obesity and insulin resistance have increased aggregation of red blood cells in their peripheral blood [9]. Imbalance of energy intake and expenditure in MAFLD population can cause insulin resistance in numerous tissues [10].

However, the relationship between RBC and MAFLD is yet to be clarified. As a result, we performed a retrospective analysis on the data from the National Health and Nutrition Examination Survey (NHANES) 2017–2018. This study aims to explore an appropriate index of hematology to predict the onset of MAFLD.

## Materials and methods

### Study population

The National Health and Nutrition Examination Survey (NHANES) is an ongoing repeated cross-sectional study at the Centers for Disease Control and Prevention (CDC) conducted by the National Center for Health Statistics (NCHS). The NHANES program was approved by the NCHS Ethics Review Board and all participants provided written informed consent. All NHANES data and information are publicly available at https://www.cdc.gov/nchs/nhanes/. Data from NHANES that were collected during 2017–2018 were included in this analysis. The participants provided their written informed consent to participate in this study. Ethical review and approval was not required for the study on human participants in accordance with the local legislation and institutional requirements.

We evaluated individuals over the age of 18 who had complete liver elastography measurements. Participants with incomplete test results or who were ineligible for hepatic elastography were eliminated from the study. Those with missing data on body mass index, RBC and important biochemistry were also excluded, leaving 4477 subjects for the analysis.

### RBCs and other laboratory measurements

RBC, Hemoglobin (Hb) and white blood cell count (WBC) was measured on venous blood in the mobile examination centers using the Beckman Coulter DxH 800 instrument. Alanine Aminotransferase (ALT), aspartate aminotransferase (AST), γ-glutamyl transferase (GGT), cholesterol (TC), Triglycerides (TG) and uric acid (SUA) were measured using the Roche Cobas 6000 chemistry analyzer (Cobas 6000). Direct high-density lipoprotein (HDL) was processed, stored, and shipped to the University of Minnesota, Minneapolis, MN for analysis. HbA1c was measured on whole blood using Trinity Biotech Boronate Affinity HPLC. In addition, insulin resistance was calculated using the homeostasis model assessment of IR (HOMA-IR) as described by Matthews et al [11]: Fasting plasma glucose (mmol/L) *Fasting insulin (μU/mL)/22.5. HOMA-IR was divided into four categories to distinguish low, middle, high and missing data.

### Measurements of other covariates

Demographic covariates included age, sex, race/ethnicity, poverty-income ratio(PIR) and smoking status which was obtained by self-report during the interview. Smoking status was categorized into current smokers, former smokers, or never smokers, determined by the participants’ answers of two questions in “Smoking-Cigarette Use” questionnaire–a) Do you now smoke cigarettes? b) Have you smoked at least 100 cigarettes in your entire life? If they didn’t smoke cigarettes now but had ever smoked at least 100 cigarettes, they were defined as former smokers. Sociodemographic characteristics were collected using a standardized self-administered questionnaire. Weight, height, and waist circumference (WC) were measured by well-trained health technologists according to the anthropometry procedure manual. Body mass index (BMI) was calculated as weight (kg) divided by height squared (m2). Blood pressure was averaged over three measurements of systolic and diastolic pressure. In addition, we defined hypertension as mean systolic blood pressure ≥140 mmHg or diastolic blood pressure ≥90 mmHg or currently taking antihypertensive medication according to self-report questionnaire. Diabetes were defined as hemoglobin A1c≥6.5%, having either diagnosed diabetes by doctor by self-report questionnaire.

### Outcome definitions

In the 2017–2018 cycle, vibration-controlled transient elastography was performed by NHANES technicians after a 2-day training program with an expert technician, using the FibroScan^®^ model 502 V2 Touch (Echosens, Paris, France) equipped with a medium (M) and extra-large (XL) probes. All participants aged 12 years and over were eligible except for those who were currently pregnant, unable to lie down, or had an implanted electronic medical device, had lesions where measurements would be taken. Only patients with complete exams (10 or more complete stiffness (E) measures, fasting time of at least 3 hours, and a liver stiffness interquartile range / median <30%) were included in the present analysis.

We defined MAFLD as the presence of metabolic risk factors in the setting of hepatic steatosis, including individuals with other concomitant liver diseases and significant alcohol consumption based on the diagnostic criteria proposed by an international expert panel. MAFLD was diagnosed as the presence of hepatic steatosis with≥1 of the following: i) overweight or obese (body mass index≥25 kg/m2), ii) diabetes mellitus, iii) at least 2 metabolic risk abnormalities. Metabolic risk abnormalities consisted of i) waist circumference≥102 cm for men and≥88 cm for women, ii) blood pressure ≥130/85mmHg or specific drug treatment, iii) fasting plasma triglycerides ≥150 mg/dl or specific drug treatment, iv) plasma HDL-cholesterol <40 mg/dl for men and <50 mg/dl for women or specific drug treatment, v) prediabetes (fasting glucose 100-125mg/dl or hemoglobin A1c 5.7%-6.4%, vi) homeostasis model assessment of insulin resistance score≥2.5, vii) plasma high-sensitivity C-reactive protein level >2 mg/L. Presence of hepatic steatosis was defined by a median Controlled Attenuation Parameter (CAP) ≥ 274 dB/m, a cut-off that yielded 90% sensitivity in distinguishing S0 from S1-S3 in a recent study by Eddowes et al [12].

### Statistic analysis

The variables were presented in two forms. Continuous variables with normal distribution were expressed as mean± standard deviation. Categorical variables are expressed as numbers and weighted proportions. One-way analysis of variance (ANOVA) was used to assess continuous variables, while the chi-squared test was used to compare categorical variables.

Because RBCs levels were significantly greater in males than in females, sex-specific RBC quartiles were generated, and all data were evaluated separately in males and females. The odds ratios (ORs) and 95% confidence intervals (CIs) of the connection between RBCs and the probability of MAFLD were calculated using logistic regression models. RBCs were divided into quartiles and the ORs with 95%CI were calculated across the quartiles. Potential confounding factors were gradually included in three models with increasing degrees of adjustment. We selected these cofounders based on their associations with the outcomes of interest or a change in effect estimate of more than 10%. Model1 was adjusted with age, race and BMI. Model 2 was fully-adjusted with age, race, BMI, diabetes mellitus, hypertension, HbA1c, HDL, SUA, TG, Hb, WBC, HOMA-IR and Smoking status. In addition, the threshold effect of RBC on MAFLD risk was explored using piecewise linear regression according to the smoothing plot.

Additionally, we ran two sensitivity analysis. Firstly, We conducted the modeling by taking the potential cofounder of ALT, SBP into account. To fill in missing data, we used multiple imputations of missing data. Secondly, we divided laboratory data into tertiles and made another logistic regression model.

All analyses were conducted using R version 3.4.3 (http://www.R-project.org) and EmpowerStats software (http://www.empowerstat.com) of NHANES.

## Results

### Clinical characteristics of study participants

The final analysis comprised 4477 patients in total, 2282 females and 2195males. Mean RBCs was higher in males(5.0± 0.5) *10^6^/μL than females (4.5±0.4) *10^6^/μL. The prevalence of MAFLD was 48.8% in males and 38.9% in females. Participant characteristics are shown in Tables 1 and 2 based on the RBC count quartiles for each gender. Participants with greater RBC quantiles had higher BMI, waist circumstance, TC, TG, ALT, and controlled attenuated parameter values than Q1 participants, and had lower HDL in both genders. All individuals were divided into quartiles based on their RBC levels, which were stratified by sex, to examine the relationship between RBC count and the risk of MAFLD. According to the RBC quartiles of the participants, the percentage of MAFLD gradually increased for both genders: 31.6%, 34.0%, 41.3%, 49.0% for females, and 43.2%, 44.7%, 49.7%, 57.2% for males in Q1, Q2, Q3, and Q4, respectively.

**Table 1:** Basic characteristics of participants according to quartiles of RBC count in females.

RBCs	Q1(≤4.26)	Q2(4.27–4.52)	Q3(4.53–4.78)	Q4(≥4.79)	P-value
N	564	573	557	588	
Age(years)	53.4 ± 18.7	47.8 ± 18.0	48.0 ± 17.5	49.4 ± 18.0	<0.001
Race					<0.001
Mexican American	56 (9.9%)	94 (16.4%)	87 (15.6%)	81 (13.8%)	
Other Hispanic	64 (11.3%)	49 (8.6%)	56 (10.1%)	64 (10.9%)	
Non-Hispanic White	186 (33.0%)	208 (36.3%)	191 (34.3%)	195 (33.2%)	
Non-Hispanic Black	170 (30.1%)	122 (21.3%)	108 (19.4%)	127 (21.6%)	
Other Race	88 (15.6%)	100 (17.5%)	115 (20.6%)	121 (20.6%)	
BMI (kg/m2)	28.9 ± 7.6	29.4 ± 8.0	30.6 ± 7.9	32.0 ± 8.3	<0.001
WAIST (cm)	95.6 ± 16.8	96.4 ± 17.3	99.6 ± 17.8	102.7 ± 17.8	<0.001
PIR					0.88
<1.3	68 (12.1%)	76 (13.3%)	76 (13.6%)	68 (11.6%)	
1.3–1.85	162 (28.7%)	159 (27.7%)	139 (25.0%)	158 (26.9%)	
>1.85	77 (13.7%)	68 (11.9%)	71 (12.7%)	75 (12.8%)	
Missing	257 (45.6%)	270 (47.1%)	271 (48.7%)	287 (48.8%)	
HbA1c (%)	5.7 ± 0.8	5.7 ± 0.9	5.8 ± 0.9	6.1 ± 1.4	<0.001
HDL (mg/dL)	60.3 ± 17.1	58.1 ± 14.9	57.0 ± 15.1	54.5 ± 14.4	<0.001
AST (IU/L)	21.3 ± 17.5	19.3 ± 9.3	20.5 ± 11.4	20.2 ± 10.6	0.07
ALT (IU/L)	17.7 ± 21.5	17.8 ± 12.0	19.3 ± 15.2	20.2 ± 14.9	0.022
GGT (IU/L)	29.3 ± 47.8	23.9 ± 32.4	24.9 ± 29.0	27.2 ± 29.5	0.046
TC (mg/dL)	186.4 ± 38.6	186.7 ± 37.9	191.7 ± 38.0	196.0 ± 43.4	<0.001
TG (mg/dL)	119.5 ± 72.1	122.7 ± 83.8	131.1 ± 76.4	139.2 ± 84.1	<0.001
SUA (μmol/L)	4.9 ± 1.5	4.6 ± 1.2	4.9 ± 1.2	5.1 ± 1.3	<0.001
SBP (mmHg)	127.4 ± 23.4	122.0 ± 20.2	125.0 ± 20.6	127.5 ± 20.9	<0.001
WBC (10^3^/μL)	7.5 ± 16.7	7.2 ± 2.1	7.4 ± 2.1	7.8 ± 2.2	0.616
RBC (10^6^/μL)	4.0 ± 0.2	4.4 ± 0.1	4.6 ± 0.1	5.1 ± 0.3	<0.001
Hb(g/μL)	12.3 ± 1.2	13.1 ± 1.0	13.6 ± 1.0	14.0 ± 1.3	<0.001
Smoking (%)					0.106
Never	371 (65.8%)	411 (71.7%)	398 (71.5%)	398 (67.7%)	
Past smoker	111 (19.7%)	87 (15.2%)	79 (14.2%)	93 (15.8%)	
Current smoker	82 (14.5%)	75 (13.1%)	80 (14.4%)	97 (16.5%)	
Diabetes(%)					0.003
No	464 (82.3%)	499 (87.1%)	463 (83.1%)	464 (78.9%)	
Yes	100 (17.7%)	74 (12.9%)	94 (16.9%)	124 (21.1%)	
Hypertension(%)					<0.001
No	292 (51.8%)	371 (64.7%)	347 (62.3%)	331 (56.3%)	
Yes	272 (48.2%)	202 (35.3%)	210 (37.7%)	257 (43.7%)	
HOMA-IR					<0.001
Low(<1.92)	112(19.9%)	98 (17.1%)	86(15.4%)	71(12.1%)	
Middle(1.92–3.83)	76(13.5%)	90(15.7%)	96(17.2%)	104(17.7%)	
High(>3.83)	61(10.8%)	68(11.9%)	99(17.8%)	139(23.6%)	
Missing	315(55.9%)	317(55.3%)	276(49.6%)	274(46.6%)	
Presence of MAFLD(%)					<0.001
No	386 (68.4%)	378 (66.0%)	327 (58.7%)	300 (51.0%)	
Yes	178 (31.6%)	195 (34.0%)	230 (41.3%)	288 (49.0%)	
CAP (dB/m)	244.5 ± 57.7	252.2 ± 59.0	263.8 ± 60.9	271.6 ± 62.4 <0.001	<0.001

*Data were presented as mean ± standard error for continuous variables and n (%) for categorical variables.

** ALT, AST, GGT, HDL, SUA, TG, HbA1c, HDL, WBC were logarithmic transformed before analysis.

***Abbreviations: PIR, ratio of family income to poverty; BMI, body mass index; ALT, aminotransferase; AST, aspartate aminotransferase; GGT, γ-glutamyl transferase; TC, total cholesterol; TG, Triglycerides; SUA, serum uric acid; SBP, systolic blood pressure; WBC, white blood cell count; RBC, red blood cell count; Hb, hemoglobin; CAP, Median controlled attenuated parameter; HOMA-IR, homeostasis model assessment- insulin resistance.

**Table 2:** Basic characteristics of participants according to quartiles of RBC count in males.

RBC quartiles	Q1(≤4.67)	Q2(4.68–4.97)	Q3(4.98–5.26)	Q4 (≥5.27)	P-value
N	535	548	551	561	
AGE (years)	61.0 ± 16.7	50.5 ± 17.0	46.2 ± 18.0	42.5 ± 17.5	<0.001
Race					<0.001
Mexican American	57 (10.7%)	66 (12.0%)	94 (17.1%)	102 (18.2%)	
Other Hispanic	48 (9.0%)	43 (7.8%)	48 (8.7%)	53 (9.4%)	
Non-Hispanic White	203 (37.9%)	212 (38.7%)	196 (35.6%)	163 (29.1%)	
Non-Hispanic Black	145 (27.1%)	119 (21.7%)	108 (19.6%)	106 (18.9%)	
Other Race	82 (15.3%)	108 (19.7%)	105 (19.1%)	137 (24.4%)	
BMI (kg/m2)	28.4 ± 6.7	28.7 ± 6.2	29.8 ± 6.5	30.2 ± 6.5	<0.001
WAIST (cm)	101.5 ± 15.9	101.3 ± 16.7	102.6 ± 16.7	103.4 ± 16.8	0.124
PIR					0.539
<1.3	70 (13.1%)	67 (12.2%)	75 (13.6%)	67 (11.9%)	
1.3–1.85	143 (26.7%)	136 (24.8%)	129 (23.4%)	121 (21.6%)	
>1.85	73 (13.6%)	85 (15.5%)	72 (13.1%)	77 (13.7%)	
Missing	249 (46.5%)	260 (47.4%)	275 (49.9%)	296 (52.8%)	
HbA1c (%)	6.0 ± 1.1	5.8 ± 1.1	5.8 ± 1.1	5.8 ± 1.2	<0.001
HDL (mg/dL)	50.9 ± 16.3	49.2 ± 14.0	47.5 ± 11.9	45.4 ± 11.4	<0.001
AST (IU/L)	23.8 ± 17.1	23.4 ± 12.4	23.5 ± 10.9	24.8 ± 13.4	0.315
ALT (IU/L)	21.7 ± 16.2	25.0 ± 16.7	27.0 ± 16.4	31.9 ± 22.3	<0.001
GGT (IU/L)	38.9 ± 59.0	37.1 ± 67.2	34.5 ± 33.9	41.0 ± 46.6	0.214
TC (mg/dL)	174.7 ± 43.2	185.4 ± 39.9	185.4 ± 39.7	188.7 ± 41.6	<0.001
TG (mg/dL)	145.7 ± 121.1	151.2 ± 115.5	163.9 ± 126.6	174.6 ± 141.7	<0.001
SUA (μmol/L)	6.1 ± 1.5	6.1 ± 1.4	6.1 ± 1.4	6.2 ± 1.2	0.398
SBP (mmHg)	131.8 ± 21.7	126.1 ± 17.3	125.8 ± 15.9	126.0 ± 15.3	<0.001
WBC (10^3^/μL)	7.0 ± 3.8	7.1 ± 2.1	7.3 ± 2.0	7.5 ± 2.2	0.006
RBC (10^6^/μL)	4.3 ± 0.3	4.8 ± 0.1	5.1 ± 0.1	5.6 ± 0.3	<0.001
Hb(g/μL)	13.5 ± 1.3	14.7 ± 0.8	15.2 ± 0.8	15.8 ± 1.1	<0.001
Smoking (%)					<0.001
Never	198 (37.0%)	257 (46.9%)	301 (54.6%)	326 (58.1%)	
Past smoker	223 (41.7%)	161 (29.4%)	146 (26.5%)	137 (24.4%)	
Current smoker	114 (21.3%)	130 (23.7%)	104 (18.9%)	98 (17.5%)	
Diabetes(%)					<0.001
No	367 (68.6%)	452 (82.5%)	461 (83.7%)	465 (82.9%)	
Yes	168 (31.4%)	96 (17.5%)	90 (16.3%)	96 (17.1%)	
Hypertension(%)					<0.001
No	227 (42.4%)	314 (57.3%)	348 (63.2%)	363 (64.7%)	
Yes	308 (57.6%)	234 (42.7%)	203 (36.8%)	198 (35.3%)	
HOMA-IR					<0.001
Low(<1.92)	92(17.2%)	106(19.3%)	84(15.2%)	70(12.5%)	
Middle (1.92–3.88)	67(12.5%)	85(15.5%)	93(16.9%)	107(19.1%)	
High(>3.88)	62(11.6%)	74(13.5%)	86(15.6%)	131(23.4%)	
Missing	314(58.7%)	283(51.6%)	288(52.3%)	253(45.1%)	
Presence of MAFLD (%)					<0.001
No	304 (56.8%)	303 (55.3%)	277 (50.3%)	240 (42.8%)	
Yes	231 (43.2%)	245 (44.7%)	274 (49.7%)	321 (57.2%)	
CAP (dB/m)	266.6 ± 64.1	271.3 ± 63.7	276.3 ± 63.9	282.1 ± 65.7	<0.001

*Data were presented as mean ± standard error for continuous variables and n (%) for categorical variables.

**Abbreviations: PIR, ratio of family income to poverty; BMI, body mass index; ALT, aminotransferase; AST, aspartate aminotransferase; GGT, γ-glutamyl transferase; TC, total cholesterol; TG, Triglycerides; HbA1c, glycosylated hemoglobin; HDL, high-density lipoprotein; SUA, serum uric acid; SBP, systolic blood pressure; WBC, white blood cell count; RBC, red blood cell count; Hb, hemoglobin; CAP, Median controlled attenuated parameter; HOMA-IR, homeostasis model assessment- insulin resistance.

### Association between RBCs and MAFLD

Binary logistic regression analysis was applied to further delineate the relationship between RBCs and MAFLD. As shown in Table 3, in the female population, we found that although RBCs were positively associated with MAFLD in non-adjusted, model1 and 2, the result was not significant. When RBCs were assessed in quartiles, although there is a remarkable rising trend in non-adjusted and model1(P for trend<0.001), the trend disappeared in model 2. The result was quite different in males. Although in the multivariate regression models, for every one unit increase in the RBCs, the risk of MAFLD was not significantly associated with RBCs (OR = 1.2,95%CI = 0.9,1.6 P = 0.10). But when RBC was assessed in quantiles, in the fully adjusted model, the presence of MAFLD showed a rising trend as the RBC quantiles increased (P for trend = 0.046). The adjusted ORs(95%CI) for the highest quartile(Q4) was 1.5(1.0, 2.3) in male.

**Table 3:** Association between RBC count and MAFLD status.

	MAFLD		
	Non-adjusted	Model I*	Model II**
Female			
RBC per unit increase	2.0 (1.6, 2.4)	1.6 (1.3, 2.1)	1.2 (0.9, 1.6)
Q1	1	1	1
Q2	1.1 (0.9, 1.4)	1.1(0.9, 1.5)	1.1 (0.8, 1.5)
Q3	**1.5 (1.2, 1.9)** ***	**1.4(1.1, 1.9)** ***	1.1 (0.8, 1.6)
Q4	**2.1 (1.6, 2.6)** ***	**1.6(1.2,2.2)** ***	1.1 (0.8, 1.6)
P for trend	<0.001	<0.001	0.439
Male			
RBC per unit increase	1.5 (1.3, 1.8)	1.6 (1.3, 2.0)	1.3 (1.0,1.8)
Q1	1	1	1
Q2	1.1 (0.8, 1.4)	1.2 (0.9, 1.7)	1.1 (0.8, 1.6)
Q3	**1.3 (1.0, 1.7)** ***	1.3 (1.0, 1.8)	1.1 (0.8, 1.7)
Q4	**1.8 (1.4, 2.2)** ***	**2.0 (1.5, 2.7)** ***	**1.5 (1.0, 2.3)** ***
P for trend	<0.001	<0.001	0.046

*Model1: Adjusted with Age, Race and BMI.

**Model2: Model1+ diabetes mellitus, hypertension, HbA1c, HDL, SUA, TG, Hb, WBC, HOMA-IR, Smoking status.

***P value <0.05.

ALT, AST, GGT, HDL, TG, HbA1c, HDL, WBC were logarithmic transformed before analysis.

### Non-linear relationship between RBCs and MAFLD in males

**Fig 1:**
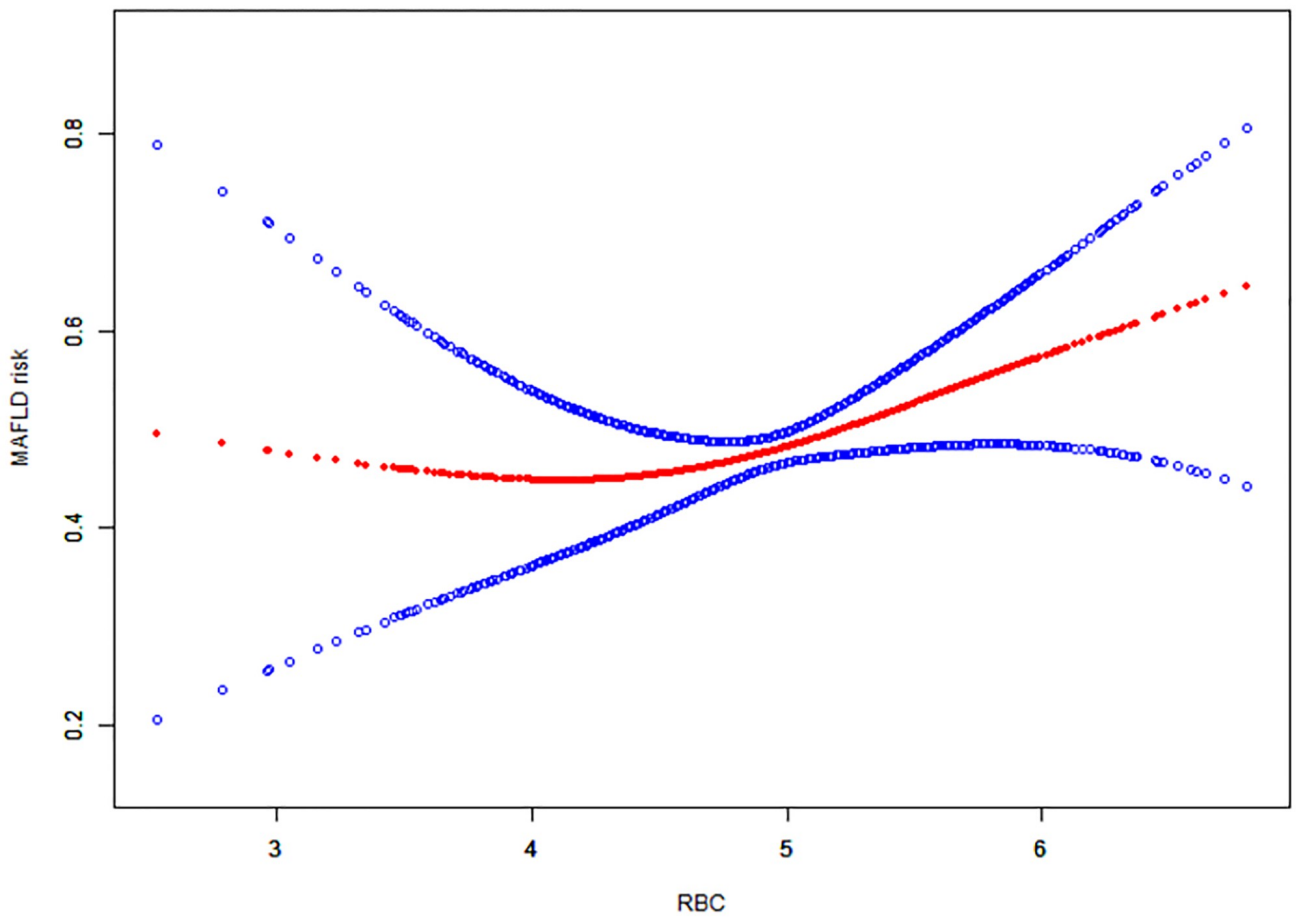
Association between RBCs and occurrence of MAFLD in male. The solid red line represents the smooth curve fit between variables. Blue bands represent the 95% confidence interval from the fit. Models were adjusted for age, race, BMI, diabetes mellitus, hypertension, HbA1c, HDL, SUA, TG, Hb, WBC, HOMA-IR, Smoking status.

To verify the linear trend between RBCs and MAFLD, we established GAM to fit the association between RBCs as a continuous variable and MAFLD by the cubic spline smoothing technique. The linear trend was observed in the female population, but the correlation was not significant as shown in S2 Fig. The smooth curve through the analysis of GAM showed that RBCs had a non-linear relationship with the occurrence of MAFLD (adjusted for age, race, BMI, HbA1c, HDL, SUA, TG, Hb, WBC and Smoking status) in the male population (Fig 1). We compared the linear regression model and two-stage linear regression to analyze the relationship between them. The P value for the log-likelihood ratio was less than 0.05. The occurrence of MAFLD increased with an increase in RBCs when RBCs > 4.2 (OR = 1.5 95%CI = 1.0, 2.0 P = 0.025) while no association was found when RBCs were lower than 4.2 as shown in Table 4.

**Table 4:** Threshold effect analysis of RBCs on MAFLD risk using piecewise liner regression in male.

	MAFLD risk (OR 95%CI)	P value
Fitting model by standard linear regression	1.3 (1.0, 1.8)	0.099
Fitting model by two-piecewise linear regression inflection point		
≤4.2	0.4 (0.1, 1.2)	0.106
>4.2	1.5 (1.0, 2.0)	0.027
P for log likelihood ratio test		0.029

*Adjusted variables were AGE, RACE, BMI, diabetes mellitus, hypertension, HbA1c, HDL, SUA, TG, Hb, WBC, HOMA-IR, Smoking status.

**Abbreviations: BMI, body mass index; HbA1c, glycosylated hemoglobin; HDL, high-density lipoprotein; TG, Triglycerides; SUA, serum uric acid; WBC, white blood cell count; Hb, hemoglobin.

### Sensitivity analysis

We made two sensitivity analysis. Firstly, we further included ALT and SBP into consideration based on Model2. Imputed data analysis was conducted and the result was shown in S3 Table. Although the upward trend disappeared (P for trend0.07), the adjusted ORs(95%CI) for the highest quartile(Q4) was 1.5(1.0,2,2) P<0.05. Secondly, multivariant logistic regression was conducted when HbA1c, HDL, SUA, TG, Hb, WBC and HOMA-IR were fitted in tertile categories. The covariate screening method was the same as above and the category boundaries were described in the footnote of S4 Table. The result was similar as above. The adjusted ORs(95%CI) for the highest quartile(Q4) was 1.5(1.0, 2.2) in male and P for trend was 0.043.

## Discussion

Multivariate analysis result showed that RBC was associated with a greater risk of MAFLD, an association independent of age, race, BMI, and other relevant factors. When RBC were divided into quartiles, the adjusted odds ratio (OR) and 95% confidence intervals (Cis)for the highest RBC quartile was 1.5(1.0,2.3) for male and 1.1 (0.8, 1.6) for female respectively while the upward trend was not significant for female. As for male, a non-linear relationship was discovered between RBC and MAFLD, with a RBC threshold of 4.2*10^6^/μL.

A meta-analysis found that MAFLD prevalence was 50.7% among overweight or obese adults in general population [13]. Another meta-analysis reported the prevalence of 5.37% (95% CI 4.36% to 6.59%) and 29.78% (95% CI 26.06% to 33.79%) of lean and nonobese individuals, respectively [14]. MAFLD criteria is more superior over NAFLD for identifying patients under high risk of hepatic fibrosis, atherosclerotic cardiovascular disease and chronic kidney disease. In patients with MAFLD, even mild alcohol consumption may cause worsening of hepatic fibrosis measures indicators [15–17]. As a result, MAFLD was supported by over 1000 signatories representative of multiple stakeholders as it more accurately reflects the underlying pathogenesis of the disease than NAFLD [18].

Our results have some similarities with previous studies. According to Wang et al, RBC is independently linked to a greater risk of developing fatty liver [19]. However, other potential factors including hemoglobin and uric acid were not adequately adjusted in their study. Mardi suggested that increased erythropoiesis and subclinical inflammation could be part of the Mets [20]. Using a longitudinal cohort design, Zhong et al. discovered that RBC is not only connected with the incidence of NAFLD but also the severity of incident NAFLD development in Chinese population [21]. Kotani confirmed that red blood cell count is an indicator related to Mets and its components, contrary to Kim’s study on Koreans with no relationship discovered between them [22,23]. In this study, RBC was significantly associated with MAFLD in males, with no such trendin females. This gender-specific result is somehow similar to previous findings that Hb concentration is associated with NAFLD and MS in men [24,25]. The relationship between RBC and MAFLD remains unclear. In our analysis, specific information on women’s health, such as menopause status or the use of hormone replacement therapy, was not assessed. And this may help explain these different findings. It has been demonstrated that low-dose estradiol-based hormone can lower liver enzyme levels and may be protective against NAFLD [26,27].

One of the key mechanisms in the emergence of MAFLD is insulin resistance. Insulin resistance is the common pathophysiological basis of metabolic dysfunctions including obesity, type 2 diabetes mellitus, hypertension, dyslipidemia, and metabolic syndrome [10]. Barbieri et al. provided in vivo evidence of a relationship between insulin resistance and red blood cell count [28]. Actually, human insulin has a synergistic effect with erythropoietin on stimulating the proliferation of the erythroid colonies [29,30]. Several studies also revealed that insulin resistance is associated with an enhanced degree of erythrocyte aggregation [9,31]. Secondly, As a a powerful sphingolipid mediator, sphingosine 1-phosphate (S1P)controls a variety of physiological responses, including cell migration, differentiation, apoptosis, lymphocyte trafficking, and inflammation [32]. Persuasive research showed that erythrocytes are the primary source of plasma S1P,which has been demonstrated as an intracellular biolipid that responds to tissue hypoxia by boosting erythrocyte glycolysis and oxygen delivery [33]. While NAFLD can create hypoxia microenvironment in which lipids accumulation in the blood vessels and the liver leads to a limited blood supply to hepatocytes, indicating that S1P may play a role in it [34]. S1P has also been shown to have a relationship with insulin resistance, hyperlipidemia and inflammation [32]. It was reported that SphK1/S1P/S1P2R pathway activation can inhibit the feedback loop of insulin secretion and sensitivity [35]. Experimental animal research and human investigations both found a positive correlation between plasma S1P and LDL cholesterol, demonstrating the significant function of S1P in the setting of hyperlipidemia [36]. Moreover, inflammation has a vital role in the progression of fatty liver disease. According to several reports, S1P activates S1PR2 to prevent macrophages from migrating to the area of inflammation [33]. Importantly, it has been discovered that NAFLD has a modified lipidomic profile, implying that modifications in the quantity and caliber of lipids in the erythrocyte membrane may have an impact on the pathogenesis and development of steatosis or steatohepatitis [37]. Furthermore, arelationship between membrane fatty acid compositions and insulin resistance was also discovered [38].

Based on the new concept of MAFLD, we explored for the first time the relationship between RBC and MAFLD. However, there still existed some limitations in this study. Firstly, although we adjusted for several important covariates, other potential factors such as physical activities, drug used and incompleteness of insulin resistance which was related to MAFLD may introduce bias. Additionally, w sex hormones were not included as covariates, probably leading to bias between sexes. Secondly, the diagnosis of hepatic steatosis was based on liver ultrasound transient elastography and there were some debates concerning the cut-off value of median Controlled Attenuation Parameter for diagnosing MAFLD, hence the diagnostic gold standard remains liver biopsy. Thirdly, it was challenging to determine a cut-off value for predicting MAFLD due to the relatively minor differences between RBC. Therefore, additional prospective research is still required to elucidate the detailed relationship between RBC and MAFLD.

## Conclusions

We demonstrated that elevated RBC is associated with the higher risk of MAFLD in males. This positive relationship was not significant in females after full adjustment. This finding provided novel evidence indicating that red blood cell count might be a potential biomarker for MAFLD in males.

## Supporting information

S1 Fig Flow chart of patient cohort. (TIF)

S2 Fig Association between RBCs and occurrence of MAFLD in female. (TIF)

S1 Table The collinearity screening of baseline characteristics. (DOCX)

S2 Table The adjusting roles of potential confounders on the estimates of RBCs on MAFLD risk. (DOCX)

S3 Table Association between RBC count and MAFLD status. (DOCX)

S4 Table Association between RBC count and MAFLD status. (DOCX)

S1 Data (CSV)

S2 Data (CSV)
