# Supplementary figures and images for: Associations between red blood cell count and metabolic dysfunction-associated fatty liver disease(MAFLD)
Source: PLoS One. 2022 Dec 27;17(12):e0279274. doi: 10.1371/journal.pone.0279274 (PMC9794081; doi:10.1371/journal.pone.0279274)

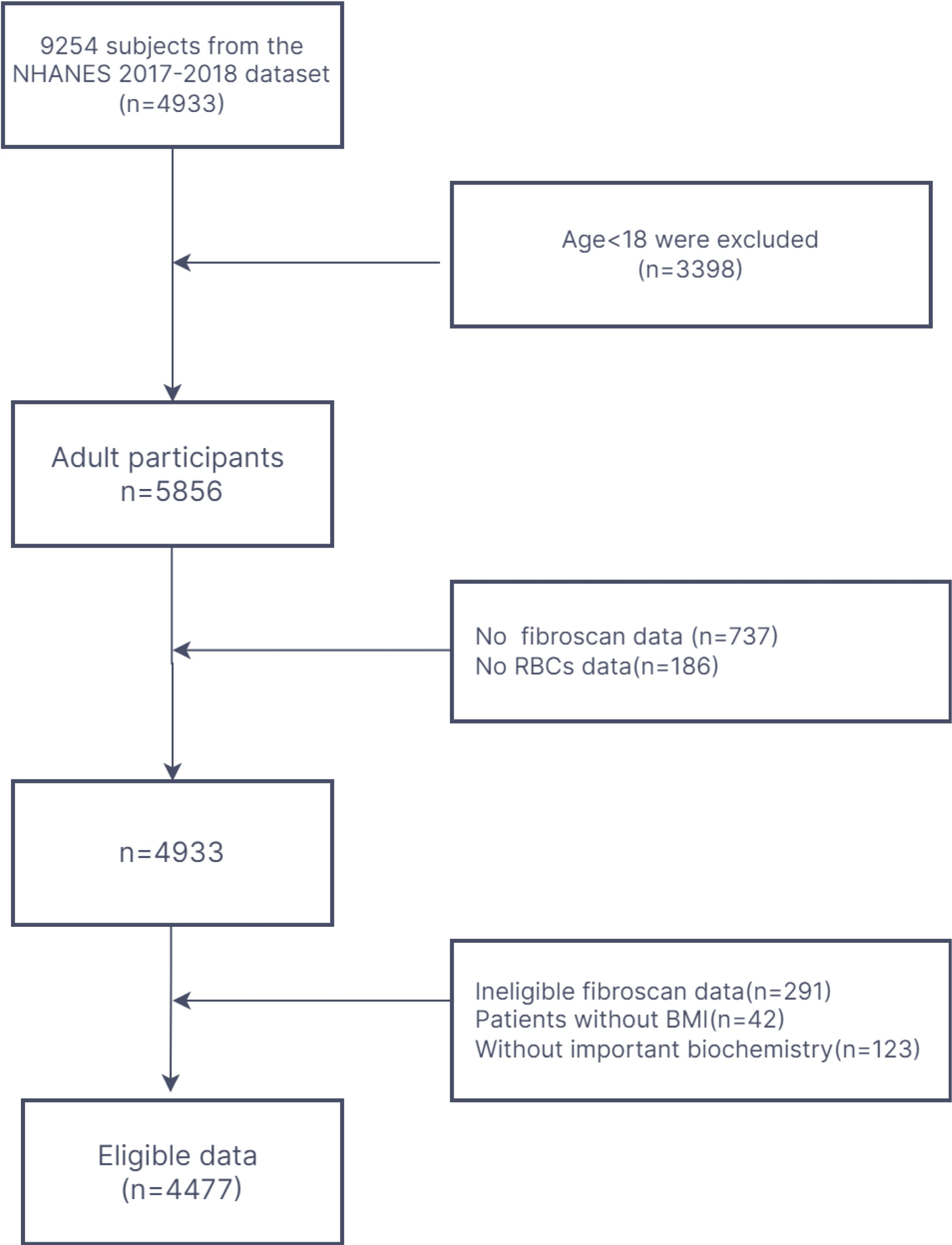

Supplement: S1 Fig — (TIF) [file pone.0279274.s001.tif]

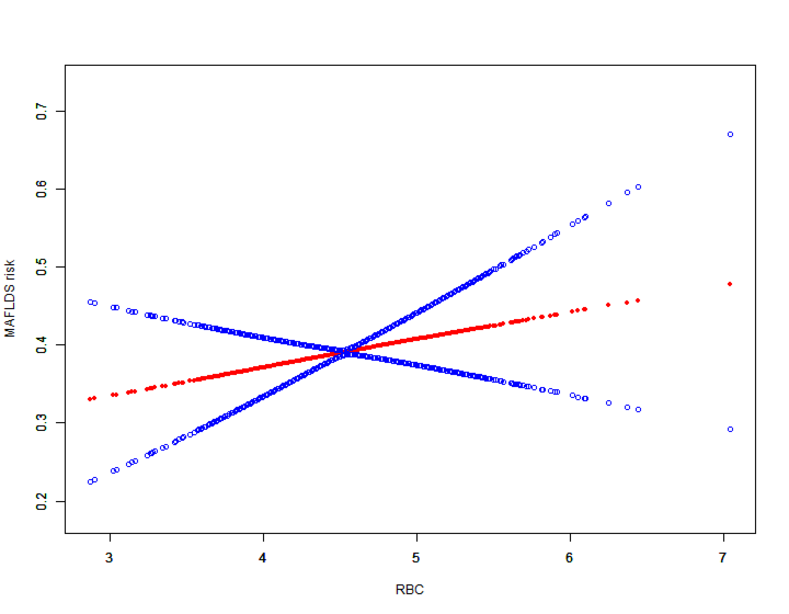

Supplement: S2 Fig — (TIF) [file pone.0279274.s002.tif]
