# Supplementary material for: Associations between red blood cell count and metabolic dysfunction-associated fatty liver disease(MAFLD)
Source: PLoS One. 2022 Dec 27;17(12):e0279274. doi: 10.1371/journal.pone.0279274 (PMC9794081; doi:10.1371/journal.pone.0279274)
Supplement: S1 Table — (DOCX) [file pone.0279274.s003.docx]

**Supporting information**

**Table1. The collinearity screening of baseline characteristics**

| Characteristics | Variance inflation factor |
| --- | --- |
| RBC | 2.3 |
| Gender | 1.9 |
| Age | 1.8 |
| Race | 1.1 |
| PIR | 1 |
| BMI | 1.3 |
| Waist | NA |
| TC | 1.5 |
| SUA | 1.4 |
| Smoking status | 1.1 |
| HbA1c | 2.4 |
| WBC | 1.3 |
| HDL | 2.1 |
| TG | 2.3 |
| Hb | 2.4 |
| SBP | 1.7 |
| Diabetes | 2.3 |
| Hypertension | 1.9 |
| HOMA-IR | 1.2 |

Abbreviations: PIR, ratio of family income to poverty; BMI, body mass index; TC, total cholesterol; TG, Triglycerides; HbA1c, glycosylated hemoglobin; HDL, high-density lipoprotein; SUA, serum uric acid; SBP, systolic blood pressure; WBC, white blood cell count; RBC, red blood cell count; Hb, hemoglobin; HOMA-IR, homeostasis model assessment of insulin resistance
