# Supplementary material for: Associations between red blood cell count and metabolic dysfunction-associated fatty liver disease(MAFLD)
Source: PLoS One. 2022 Dec 27;17(12):e0279274. doi: 10.1371/journal.pone.0279274 (PMC9794081; doi:10.1371/journal.pone.0279274)
Supplement: S2 Table — (DOCX) [file pone.0279274.s004.docx]

**Table2 The adjusting roles of potential confounders on the estimates of RBCs on MAFLD risk**

| +/- covariates | Basic model | Complete model | Selected covariates |
| --- | --- | --- | --- |
| Initial regression coefficient of RBCs | 0.6045 | 0.1749 |  |
| Gender | 0.5200 * | 0.2112 * | Yes |
| Age | 0.8026 * | 0.0675 * | Yes |
| Race | 0.5915 | 0.1170 * | Yes |
| PIR | 0.6023 | 0.1762 |  |
| BMI | 0.5189 * | 0.3564 * | Yes |
| TC | 0.5894 | 0.1813 |  |
| SUA | 0.4442 * | 0.175 | Yes |
| Smoking status | 0.6216 | 0.1929 * | Yes |
| HbA1c | 0.6226 | 0.2376 * | Yes |
| WBC | 0.5395 * | 0.1834 | Yes |
| HDL | 0.3113 * | 0.1964 * | Yes |
| TG | 0.4100 * | 0.1411 * | Yes |
| Hb | 0.5251 * | 0.2580 * | Yes |
| SBP | 0.6503 | 0.1744 |  |
| Diabetes | 0.6841 * | 0.1674 | Yes |
| Hypertension | 0.7156 * | 0.1749 | Yes |
| HOMA-IR | 0.5267 * | 0.1953 * | Yes |

Abbreviations: PIR, ratio of family income to poverty; BMI, body mass index; TC, total cholesterol; TG, Triglycerides; HbA1c, glycosylated hemoglobin; HDL, high-density lipoprotein; SUA, serum uric acid; SBP, systolic blood pressure; WBC, white blood cell count; RBC, red blood cell count; Hb, hemoglobin; HOMA-IR, homeostasis model assessment of insulin resistance

ALT, HDL, TG, HbA1c, WBC were logarithmic transformed before analysis.
