# Supplementary material for: Associations between red blood cell count and metabolic dysfunction-associated fatty liver disease(MAFLD)
Source: PLoS One. 2022 Dec 27;17(12):e0279274. doi: 10.1371/journal.pone.0279274 (PMC9794081; doi:10.1371/journal.pone.0279274)
Supplement: S3 Table — (DOCX) [file pone.0279274.s005.docx]

**Table3** **Association between RBC count and MAFLD status.**

|  | Model 3 | |
| --- | --- | --- |
|  | Male | Female |
|  | OR(95%CI) | OR(95%CI) |
| RBCs |  |  |
| Q1 | 1 | 1 |
| Q2 | 1.1(0.8,1.6) | 1.1(0.8,1.5) |
| Q3 | 1.1(0.8,1.6) | 1.2(0.8,1.6) |
| Q4  P for trend | **1.5(1.0,2.2)**  0.07 | 1.2(0.9,1.7)  0.27 |

Model3: Adjusted with Age, Race, BMI, Diabetes mellitus, Hypertension, HbA1c, HDL, SUA, TG, Hb, WBC, Smoking status, ALT, SBP. ALT、HDL、TG、HbA1c、HDL、WBC were logarithmic transformed before analysis.
