# Supplementary material for: Associations between red blood cell count and metabolic dysfunction-associated fatty liver disease(MAFLD)
Source: PLoS One. 2022 Dec 27;17(12):e0279274. doi: 10.1371/journal.pone.0279274 (PMC9794081; doi:10.1371/journal.pone.0279274)
Supplement: S4 Table — (DOCX) [file pone.0279274.s006.docx]

Table4 Association between RBC count and MAFLD status.

|  | Model 4 | |
| --- | --- | --- |
|  | Male | Female |
|  | OR(95%CI) | OR(95%CI) |
| RBCs |  |  |
| Q1 | 1 | 1 |
| Q2 | 1.1(0.8,1.5) | 1.1(0.8,1.5) |
| Q3 | 1.1(0.8,1.6) | 1.1(0.8,1.6) |
| Q4  P for trend | **1.5(1.0,2.2)**  **0.043** | 1.2(0.8,1.7)  0.31 |

Model4：Adjusted with Age, Race, BMI, Diabetes mellitus, Hypertension, HbA1c, HDL, SUA, TG, Hb, WBC. HbA1c, HDL, SUA, TG, Hb, WBC, HOMA-IR were fitted in tertile categories before analysis.

Male: HbA1c was divided in tertiles with levels set at <5.4,5.4-5.7,>5.7. HDL was divided in tertiles with levels set at <41,41-50,>50. SUA was divided in tertiles with levels set at <5.5,5.4-6.5,>6.5. TG was divided in tertiles with levels set at <98,98-162,>162. Hb was divided in tertiles with levels set at <14.5,14.5-15.3,>15.3. WBC was divided in tertiles with levels set at <6.1,6.1-7.7,>7.7. HOMA-IR was divided in tertiles with levels set at <1.92,1.92-3.88,>3.88. Female：HbA1c was divided in tertiles with levels set at <5.4,5.4-5.7,>5.7. HDL was divided in tertiles with levels set at <49,49-61,>61. SUA was divided in tertiles with levels set at <4.2,4.2-5.2,>5.2. TG was divided in tertiles with levels set at <87,87-136,>136. Hb was divided in tertiles with levels set at <12.9,12.9-13.7,>13.7. WBC was divided in tertiles with levels set at <6.1,6.1-7.9,>7.9. HOMA-IR was divided in tertiles with levels set at <1.92,1.92-3.83,>3.83.
